# Association of Racial/Ethnic and Gender Concordance Between Patients and Physicians With Patient Experience Ratings

**DOI:** 10.1001/jamanetworkopen.2020.24583

**Published:** 2020-11-09

**Authors:** Junko Takeshita, Shiyu Wang, Alison W. Loren, Nandita Mitra, Justine Shults, Daniel B. Shin, Deirdre L. Sawinski

**Affiliations:** 1 Department of Dermatology, Perelman School of Medicine at the University of Pennsylvania, Philadelphia; 2 Department of Biostatistics, Epidemiology and Informatics, Perelman School of Medicine at the University of Pennsylvania, Philadelphia; 3 Hematology/Oncology Division, Department of Medicine, Perelman School of Medicine at the University of Pennsylvania, Philadelphia; 4 Renal-Electrolyte and Hypertension Division, Department of Medicine, Perelman School of Medicine at the University of Pennsylvania, Philadelphia

## Abstract

**Importance:**

The Press Ganey Outpatient Medical Practice Survey is used to measure the patient experience. An understanding of the patient- and physician-related determinants of the patient experience may help identify opportunities to improve health care delivery and physician ratings.

**Objective:**

To evaluate the associations between the patient experience as measured by scores on the Press Ganey survey and patient-physician racial/ethnic and gender concordance.

**Design, Setting, and Participants:**

A cross-sectional analysis of Press Ganey surveys returned for outpatient visits within the University of Pennsylvania Health System between 2014 and 2017 was performed. Participants included adult patient and physician dyads for whom surveys were returned. Data analysis was performed from January to June 2019.

**Exposures:**

Patient-physician racial/ethnic and gender concordance.

**Main Outcomes and Measures:**

The primary outcome was receipt of the maximum score for the “likelihood of your recommending this care provider to others” question in the Care Provider domain of the Press Ganey survey. Secondary outcomes included each of the remaining 9 questions in the Care Provider domain. Generalized estimating equations clustering on physicians with exchangeable intracluster correlations and cluster-robust standard errors were used to investigate associations between the outcomes and patient-physician racial/ethnic and gender concordance.

**Results:**

In total, 117 589 surveys were evaluated, corresponding to 92 238 unique patients (mean [SD] age, 57.7 [15.6] years; 37 002 men [40.1%]; 75 307 White patients [81.6%]) and 747 unique physicians (mean [SD] age 45.5 [10.6] years; 472 men [63.2%]; 533 White physicians [71.4%]). Compared with racially/ethnically concordant patient-physician dyads, discordance was associated with a lower likelihood of physicians receiving the maximum score (adjusted odds ratio [OR], 0.88; 95% CI, 0.82-0.94; *P* < .001). Black (adjusted OR, 0.73; 95% CI, 0.68-0.78; *P* < .001) and Asian (adjusted OR, 0.55; 95% CI, 0.50-0.60; *P* < .001) patient race were both associated with lower patient experience ratings. Patient-physician gender concordance was not associated with Press Ganey scores (adjusted OR, 1.00; 95% CI, 0.96-1.04; *P* = .90).

**Conclusions and Relevance:**

In this study, higher Press Ganey survey scores were associated with racial/ethnic concordance between patients and their physicians. Efforts to improve physician workforce diversity are imperative. Delivery of health care in a culturally mindful manner between racially/ethnically discordant patient-physician dyads is also essential. Furthermore, Press Ganey scores may differ by a physician’s patient demographic mix; thus, care must be taken when publicly reporting or using Press Ganey scores to evaluate physicians on an individual level.

## Key Points

**Question:**

Is patient-physician racial/ethnic or gender concordance associated with the patient experience as measured by scores on the Press Ganey Outpatient Medical Practice Survey?

**Findings:**

In this cross-sectional analysis of 117 589 Press Ganey surveys completed for the adult outpatient practices of an urban, academic health system from 2014 to 2017, physicians among racially/ethnically discordant patient-physician dyads had significantly lower odds of receiving the maximum patient experience score compared with those among concordant dyads.

**Meaning:**

In this study, higher Press Ganey survey scores were associated with racial/ethnic concordance between patients and their physicians; thus, efforts to improve the patient experience among racially/ethnically discordant patient-physician dyads may be necessary to improve health care delivery.

## Teaser

This cross-sectional study evaluates the associations between the patient experience as measured by scores on the Press Ganey Outpatient Medical Practice Survey and patient-physician racial/ethnic and gender concordance.

## Introduction

The patient experience is at the heart of patient-centered medical care. There have been increasing efforts not only to measure the patient experience but also to publicly share patient ratings for individual physicians, sometimes linking patient ratings to physician employment and compensation,^1^ with the ultimate goal of motivating improvements in health care delivery. The Press Ganey (PG) Outpatient Medical Practice Survey^2^ is used by many practices to evaluate the patient experience. Despite its wide use, the associations between the patient experience and patient, physician, and clinical encounter characteristics remain unclear.

Much work has focused on patient and physician race/ethnicity or gender and their associations with the patient experience and specific aspects of the patient-physician relationship.^3,4,5,6,7,8,9,10,11,12,13,14,15,16,17,18,19,20,21^ The existing studies on this topic are quite heterogenous in terms of populations evaluated, methods used, and outcomes measured, which may, in part, contribute to mixed findings. Although some studies suggest that there are benefits associated with patient-physician racial/ethnic or gender concordance,^8,10,11,12,19^ others do not.^5,13,14,15,16,17,18,19,21^ In light of existing racial/ethnic and gender health and health care disparities,^22^ as well as increasing diversification of the US population,^23^ a better understanding of the associations between patient and physician demographics and the patient experience of health care is a necessary and important step toward our collective goal of achieving health equity. Furthermore, an evaluation of these associations is critical to quality improvement efforts and appropriate interpretation and use of patient experience ratings. Many of the existing studies of the PG survey have been limited by relatively narrow patient populations, exclusion of patient and physician characteristics that may be important determinants of the patient experience, or focus on specific medical specialties.^5,10,20,24^ We evaluated the associations between the patient experience as measured by the PG survey and patient-physician racial/ethnic and gender concordance among a diverse, urban, ambulatory patient population across all medical specialties.

## Methods

### Study Design and Data Sources

We conducted a cross-sectional analysis to evaluate the associations between the patient experience, as measured by PG scores obtained from the PG Outpatient Medical Practice Survey, and patient-physician racial/ethnic and gender concordance. Our primary data source was patient experience surveys, including the complete PG survey and components of the Clinician and Group Consumer Assessment of Healthcare Providers and Systems (CG CAHPS) surveys (eAppendix in the Supplement)^25^ for outpatient visits within the University of Pennsylvania Health System (UPHS) between July 2, 2014, and May 30, 2017. Patient and clinical encounter characteristics were obtained from the survey and the electronic medical record; physician characteristics were obtained from the University of Pennsylvania faculty database.

This study was approved by the University of Pennsylvania institutional review board. A waiver of informed consent was approved because the data were deidentified. The study is reported according to the Strengthening the Reporting of Observational Studies in Epidemiology (STROBE) reporting guideline.^26^

The PG survey evaluates 6 domains (Access, Moving Through Your Visit, Nurse/Assistant, Care Provider, Personal Issues, and Overall Assessment) with 2 to 10 Likert-type item questions per domain and 5 answer options ranging from 1 (very poor) to 5 (very good).^2^ Paper or electronic surveys were sent to all patients who received outpatient care within UPHS. Patients did not receive surveys if they declined solicitation or did not have a valid mailing or email address on file. The survey response rate during the study period was 19.9%.

### Study Population

The study included returned surveys for all adult (aged ≥18 years old) outpatient visits with nontrainee physicians within UPHS during the study period. Surveys were excluded if any of the following applied: (1) clinician lacked MD or DO credentials; (2) patient or physician age, gender, or race/ethnicity was missing; (3) patient or physician race/ethnicity other than non-Hispanic White (hereafter referred to as White), non-Hispanic Black (hereafter referred to as Black), non-Hispanic Asian (including Southeast Asians and Pacific Islanders; hereafter referred to as Asian), or Hispanic ethnicity of any race (hereafter referred to as Hispanic); or (4) the primary outcome was missing. Only the first survey for any patient-physician pair was included, resulting in 1 survey for each unique patient-physician pair (eFigure 1 in the Supplement).

### Definitions of Exposures and Covariates

#### Exposures

The primary exposures were patient-physician racial/ethnic and gender concordance. Concordance was evaluated both as a binary measure and by specific patient-physician racial/ethnic and gender pairs. Patient race/ethnicity was obtained from self-report in the CG CAHPS survey or, if unavailable, from the medical record. Patient gender was obtained from the medical record. Self-reported physician race/ethnicity and gender were obtained from the university’s faculty database. Race/ethnicity was categorized as White, Black, Asian, or Hispanic. Gender was categorized as male or female.

#### Covariates: Patient, Physician, and Clinical Encounter Variables

Other patient characteristics included age, marital status, insurance, highest education level, median annual household income of the patient’s zip code of residence, primary language, overall health, overall mental or emotional health, and level of assistance with survey completion. Highest education level, overall health, overall mental or emotional health, and level of assistance with survey completion were patient-reported from the survey. All other variables were self-reported and obtained from the medical record.

Other physician characteristics were obtained from the university’s faculty database and included self-reported age, type of medical degree, additional degrees, and faculty track and rank. Clinical encounter characteristics were obtained from the medical record and included medical specialty, visit type, location, and year.

### Definitions of Outcomes

#### Primary Outcome

The primary outcome was derived from the score for the item, “Likelihood of your recommending this care provider to others” in the Care Provider domain of the PG survey. Because the responses were highly skewed, with most scores clustered at the high values, we primarily used the top box scoring method, whereby scores were dichotomized as 5 (maximum score) vs less than 5.^2^ This method is consistent with current practices for patient experience score reporting.^10,24,27,28^ We also reported mean scores on a 1 to 5 scale.^29^

#### Secondary Outcomes

Each of the remaining 9 questions under the Care Provider domain of the PG survey served as secondary outcomes. Each secondary outcome score was dichotomized as 5 vs less than 5.

### Statistical Analysis

Patient, physician, and clinical encounter characteristics were summarized with descriptive statistics. To investigate associations between each outcome and patient-physician racial/ethnic and gender concordance, we used generalized estimating equations clustering on physicians with exchangeable intracluster correlations and cluster-robust standard errors to obtain population-averaged estimates of statistical associations. We used purposeful selection to build the multivariable regression model for the primary outcome.^30^ Patient and physician age, gender, and race were included in all models, as well as any patient, physician, or clinical encounter characteristics that were significantly associated with the primary outcome at α level of .05 in univariable analyses. For the models using binary racial/ethnic or gender concordance, physician race/ethnicity or gender, respectively, were excluded to avoid overfitting. For the regression models evaluating the associations between specific patient-physician racial/ethnic or gender pairs and the primary outcome, indicator variables for race/ethnicity or gender of the patient and physician and their interaction term (product), respectively, were included. To compare the odds of the outcome for each specific patient-physician racial/ethnic or gender pairs, we computed the odds ratios (ORs) from the linear combinations of each discordant pair from their respective final regression models. Associations between secondary outcomes and patient-physician racial/ethnic and gender concordance were evaluated using the final multivariable models for the association between the primary outcome and racial/ethnic and gender concordance, respectively. Concordant race/ethnicity or gender served as the reference in all models. *P* values were not adjusted for multiplicity for the secondary outcomes because they were exploratory in nature. Statistical analyses were performed using Stata statistical software version 15 (StataCorp). Statistical significance was determined by 2-sided *P* values at *P* < .05. Data analysis was performed from January to June 2019.

## Results

### Study Population

The total number of surveys during the study period was 246 138, representing 124 957 unique patients and 2249 unique physicians. After application of study selection criteria (eFigure 1 in the Supplement), the number of surveys was 117 589, representing 92 238 unique patients and 747 unique physicians.

### Patient, Physician, and Clinical Encounter Characteristics

Patient characteristics are summarized in Table 1. The mean (SD) age was 57.7 (15.6) years, and 37 002 patients (40.1%) were men. The racial/ethnic distribution was majority White (75 307 patients [81.6%]) followed by Black (11 759 patients [12.7%]), Asian (3087 patients [3.3%]), and Hispanic (2085 patients [2.3%]). Most patients had commercial insurance (49 537 patients [53.7%]) and reported being at least a 4-year college graduate (55 255 patients [59.9%]). The mean (SD) median annual household income was $78 834 ($31 470).

**Table 1.** Patient Characteristics

Characteristic	Unique patients, No. (%) (N = 92 238)
No. of surveys per patient	
Mean (SD)	1.27 (0.65)
Median (IQR)	1 (1-1)
Age, y	
Mean (SD)	57.7 (15.6)
Median (IQR)	60 (48-69)
Male	37 002 (40.1)
Race/ethnicity	
White	75 307 (81.6)
Black	11 759 (12.7)
Asian	3087 (3.3)
Hispanic	2085 (2.3)
Marital status	
Single	21 241 (23.0)
Married or with a partner	59 590 (64.6)
Divorced or widowed	10 987 (11.9)
Other	420 (0.46)
Insurance	
Commercial	49 537 (53.7)
Medicare	30 328 (32.9)
Medicaid	3617 (3.9)
Tricare or Veterans Affairs	589 (0.64)
Workers’ compensation	383 (0.42)
Mixed	5462 (5.9)
Other	2322 (2.5)
Education	
Up to high school or general education diploma	15 408 (16.7)
Some college	20 593 (22.3)
4-y College graduate	20 034 (21.7)
More than 4-y college graduate	35 221 (38.2)
Unknown	982 (1.1)
Median annual household income, $	
Mean (SD)	78 834 (31 470)
Median (IQR)	76 464 (56 634-97 452)
Primary language English	91 363 (99.1)
Survey assistance	
Read questions	578 (0.63)
Wrote answers	688 (0.75)
Answered for me	1294 (1.4)
Translation	115 (0.12)
Other	250 (0.27)
None	88 312 (95.7)
Unknown	1001 (1.1)
Overall health	
Poor	2807 (3.0)
Fair	12 790 (13.9)
Good	29 582 (32.1)
Very good	33174 (36.0)
Excellent	13 327 (14.4)
Unknown	558 (0.60)
Overall mental or emotional health	
Poor	1293 (1.4)
Fair	7748 (8.4)
Good	19 861 (21.5)
Very good	32 049 (34.7)
Excellent	30 765 (33.4)
Unknown	522 (0.57)

Abbreviation: IQR, interquartile range.

Physician characteristics are summarized in Table 2. The mean (SD) age was 45.5 (10.6) years, and 472 (63.2%) were men. Racial/ethnic distribution was majority White (533 physicians [71.4%]) followed by Asian (157 physicians [21.0%]), Black (34 physicians [4.6%]), and Hispanic (23 physicians [3.1%]). Most physicians had an MD degree (737 physicians [98.7%]) and were assistant professors (375 physicians [50.2%]). Patient characteristics stratified by physician race/ethnicity are summarized in eTable 1 in the Supplement.

**Table 2.** Physician Characteristics

Characteristic	Unique physicians, No. (%) (N = 747)
No. of surveys per physician	
Mean (SD)	157.4 (162.2)
Median (IQR)	114 (46-213)
Age, y	
Mean (SD)	45.5 (10.6)
Median (IQR)	43 (37-53)
Male	472 (63.2)
Race/ethnicity	
White	533 (71.4)
Black	34 (4.6)
Asian	157 (21.0)
Hispanic	23 (3.1)
Credentials	
MD	737 (98.7)
DO	10 (1.3)
Additional degree	
PhD	69 (9.2)
Other	84 (11.2)
None	594 (79.5)
Faculty track	
Clinician	91 (12.2)
Academic clinician	255 (34.1)
Clinician educator	274 (36.7)
Tenure	57 (7.6)
Other	70 (9.4)
Faculty rank	
Assistant professor	375 (50.2)
Associate professor	163 (21.8)
Professor	165 (22.1)
Other	44 (5.9)

Abbreviation: IQR, interquartile range.

Clinical encounter characteristics are summarized in Table 3. Most encounters represented return visits (75 844 encounters [64.5%]) and were for medical (40 010 encounters [34.0%]) and surgical (35 116 encounters [29.9%]) specialties.

**Table 3.** Clinical Encounter Characteristics

Characteristic	Clinical encounters, No. (%) (N = 117 589)
Specialty^a^	
Medical	40 010 (34.0)
Surgical	35 116 (29.9)
Dermatology	16 289 (13.9)
Other	26 174 (22.3)
Visit type	
New	39 428 (33.5)
Return	75 844 (64.5)
Procedure	2316 (2.0)
Location	
Main	49 051 (41.7)
Affiliated, Philadelphia	42 569 (36.2)
Satellite, Pennsylvania	22 568 (19.2)
Satellite, New Jersey	3401 (2.9)
Survey year	
2014	16 654 (14.2)
2015	37 836 (32.2)
2016	43 340 (36.9)
2017	19 759 (16.8)

^a^ Medical specialties included family practice, general internal medicine, and all internal medicine specialties. Surgical specialties included anesthesia or pain, and cardiac; colorectal; ear, nose, and throat; gastrointestinal; neurologic; oncologic; orthopedic; plastic; thoracic; trauma; transplant; urologic; and vascular surgery. The other specialty category includes neurology, obstetrics-gynecology, ophthalmology, palliative care, physical medicine and rehabilitation, psychiatry, and radiology.

### Racial/Ethnic Concordance

#### Primary Outcome

Overall, physicians in 67 504 of 77 051 (87.6%) racially/ethnically concordant patient-physician encounters received the maximum score, compared with physicians in 33 280 of 40 538 (82.1%) discordant patient-physician encounters (a 5.5-point difference). In adjusted analyses, physicians in racially/ethnically discordant patient-physician pairs were significantly less likely to receive the maximum score compared with those in concordant pairs (adjusted OR, 0.88; 95% CI, 0.82-0.94; *P* < .001).

The proportions of encounters in which physicians received the maximum score and the mean scores for encounters by specific patient-physician racial/ethnic pairs are summarized in Table 4. The distribution of scores differs by physician race/ethnicity within each patient racial/ethnic group but also differs by patient racial/ethnic group. White patients were generally most likely to provide the maximum score for their physicians across all physician racial/ethnic groups (range, 84.9% [3025 of 3562 encounters] to 87.9% [65 775 of 74 850 encounters]; mean score, 4.80 among all physicians), whereas Asian patients were the least likely to provide the maximum score for their physicians (range 70.3% [116 of 165 encounters] to 76.5% [78 of 102 encounters]; mean score, 4.61 among all physicians).

**Table 4.** Distribution of Press Ganey Scores by Patient-Physician Racial/Ethnic Pairs

Physician race/ethnicity and Press Ganey score	Patients, No. (%)				*P* value^a^
	White	Black	Asian	Hispanic	
Any race/ethnicity					
Very good	84 669 (87.4)	11 292 (78.3)	2767 (73.4)	2056 (81.5)	<.001
Good	8302 (8.6)	2307 (16.0)	715 (19.0)	305 (12.1)	
Fair	1895 (2.0)	433 (3.0)	175 (4.6)	80 (3.2)	
Poor	774 (0.8)	156 (1.1)	51 (1.4)	25 (1.0)	
Very poor	1227 (1.3)	241 (1.7)	63 (1.7)	56 (2.2)	
Overall score, mean (SD)^b^	4.80 (0.63)	4.68 (0.74)	4.61 (0.78)	4.70 (0.78)	<.001
White					
Very good	65 775 (87.9)	7757 (78.3)	1974 (73.8)	1511 (81.9)	<.001
Good	6219 (8.3)	1595 (16.1)	499 (18.6)	221 (12.0)	
Fair	1396 (1.9)	275 (2.8)	121 (4.5)	55 (3.0)	
Poor	569 (0.8)	116 (1.2)	39 (1.5)	16 (0.9)	
Very poor	891 (1.2)	159 (1.6)	43 (1.6)	42 (2.3)	
Overall score, mean (SD)^b^	4.81 (0.62)	4.68 (0.73)	4.62 (0.78)	4.70 (0.77)	<.001
Black					
Very good	3025 (84.9)	1059 (82.4)	116 (70.3)	105 (77.8)	.04
Good	358 (10.1)	149 (11.6)	40 (24.2)	22 (16.3)	
Fair	75 (2.1)	39 (3.0)	7 (4.2)	5 (3.7)	
Poor	49 (1.4)	11 (0.9)	0	2 (1.5)	
Very poor	55 (1.5)	27 (2.1)	2 (1.2)	1 (0.7)	
Overall score, mean (SD)^b^	4.75 (0.70)	4.71 (0.75)	4.62 (0.68)	4.69 (0.69)	<.001
Asian					
Very good	14 197 (85.9)	2173 (76.2)	599 (72.3)	369 (81.3)	<.001
Good	1548 (9.4)	501 (17.6)	158 (19.1)	50 (11.0)	
Fair	390 (2.4)	104 (3.6)	43 (5.2)	17 (3.7)	
Poor	138 (0.8)	21 (0.7)	11 (1.3)	7 (1.5)	
Very poor	255 (1.5)	53 (1.9)	17 (2.1)	11 (2.4)	
Overall score, mean (SD)^b^	4.77 (0.68)	4.65 (0.75)	4.58 (0.82)	4.67 (0.82)	<.001
Hispanic					
Very good	1672 (86.8)	303 (77.7)	78 (76.5)	71 (80.7)	<.001
Good	177 (9.2)	62 (15.9)	18 (17.6)	12 (13.6)	
Fair	34 (1.8)	15 (3.8)	4 (3.9)	3 (3.4)	
Poor	18 (0.9)	8 (2.1)	1 (1.0)	0	
Very poor	26 (1.3)	2 (0.5)	1 (1.0)	2 (2.3)	
Overall score, mean (SD)^b^	4.79 (0.65)	4.68 (0.69)	4.68 (0.69)	4.70 (0.75)	.009

^a^ *P* values were calculated using χ^2^ test for categorical Press Ganey scores and analysis of variance test for continuous Press Ganey scores.

^b^ Scores were calculated on a scale of 1 to 5.

Adjusted ORs for the primary outcome by specific patient-physician racial/ethnic pairs are summarized in the Figure. Among White patients, Asian physicians had lower odds of receiving the maximum score compared with White physicians (adjusted OR, 0.87; 95% CI, 0.78-0.97; *P* = .01); Black physicians also had lower odds of receiving the maximum score vs White physicians but the difference was not significant (adjusted OR, 0.79; 95% CI, 0.60-1.04; *P* = .09). The absolute difference in mean score between White and Asian physicians was 0.03, and that between White and Black physicians was 0.05. The likelihood of receiving the maximum score was similar between Hispanic and White physicians (adjusted OR, 1.02; 95% CI, 0.78-1.35; *P* = .87). Among Black patients, White (adjusted OR, 0.73; 95% CI, 0.55-0.97; *P* = .03) and Asian (adjusted OR, 0.67; 95% CI, 0.50-0.90; *P* = .01) physicians were each less likely to receive the maximum score compared with Black physicians. The OR for Hispanic physicians was not statistically significant (adjusted OR, 0.80; 95% CI, 0.56-1.16; *P* = .24). The absolute difference in mean score between Black and White physicians was 0.03, and that between Black and Asian physicians was 0.06. The ORs among Asian and Hispanic patients were not significant for any of the patient-physician pairs.

**Figure.**
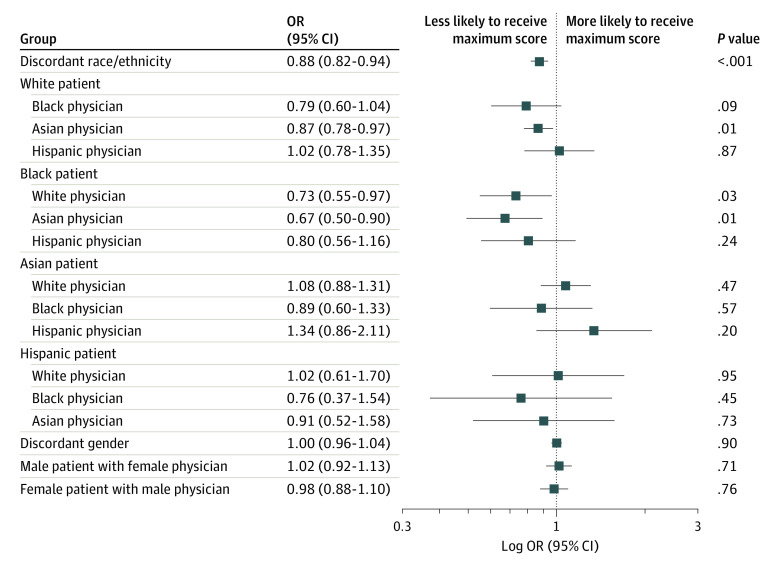
Adjusted Odds Ratios (ORs) for Maximum Press Ganey Score by Patient-Physician Racial/Ethnic and Gender Discordant Pairs Patient-physician racial/ethnic and gender concordance regression models included the following variables: patient age, gender, race/ethnicity, education, income, marital status, insurance, primary language, overall health, and mental and emotional health; physician age, gender, race/ethnicity, academic track (gender concordance only), and academic rank; and clinical encounter type, location, and specialty. Scores refer to scores on the Press Ganey Outpatient Medical Practice survey. Patient-physician concordant pairs serve as the reference.

#### Secondary Outcomes

ORs for the secondary outcomes by discordant patient-physician racial/ethnic pairs are summarized in eTable 2 in the Supplement. The ORs are similar to those for the primary outcome.

### Gender Concordance: Primary and Secondary Outcomes

Overall, physicians in 57 924 of 67 290 (86.1%) gender-concordant patient-physician encounters received the maximum score, compared with physicians in 42 860 of 50 299 (85.2%) gender-discordant patient-physician encounters. In adjusted analyses, physicians in gender discordant patient-physician pairs were equally as likely to receive the maximum score as those in concordant pairs (adjusted OR, 1.00; 95% CI, 0.96-1.04; *P* = .90). Adjusted ORs for the primary outcome by specific patient-physician gender pairs are summarized in the Figure and reveal no significant differences. Adjusted ORs for the secondary outcomes by patient-physician gender pairs are shown in eFigure 2 in the Supplement.

### Other Patient, Physician, and Clinical Encounter Characteristics

Associations between other patient, physician, and clinical encounter characteristics and the primary outcome are summarized in eTable 3 and eTable 4 in the Supplement. Patient characteristics consistently associated with higher odds of giving the maximum score in both race/ethnicity and gender concordance models include older age, male gender, married or partnered or divorced or widowed marital status, Medicare or Medicaid insurance, and better self-reported overall and mental or emotional health. Patient characteristics consistently associated with lower odds of giving the maximum score include Black (race/ethnicity concordance model, adjusted OR, 0.73; 95% CI, 0.68-0.78; *P* < .001) and Asian race (race/ethnicity concordance model, adjusted OR, 0.55; 95% CI, 0.50-0.60; *P* < .001), higher education levels, and non-English primary language. Higher physician rank (professor) was consistently associated with higher odds of receiving the highest score; older physician age was associated with lower odds of receiving the highest score. Return and procedural visits were each associated with higher odds of receiving the top score. Clinical encounters in specialties outside of medicine were associated with lower odds of receiving the highest score.

## Discussion

In this study of PG scores from patients seen in the ambulatory practices of an urban, academic medical center, we found that patient-physician racial/ethnic concordance was associated with patients’ reported experience with their physicians. Compared with physicians in racially/ethnically concordant patient-physician encounters, physicians in discordant dyads were less likely to receive the maximum patient rating. On an absolute level, there was a 5.5-point difference between the percentages of physicians receiving the highest score in discordant vs concordant dyads. Among specific patient-physician racial/ethnic dyads, relative and absolute measures showed that White patients who saw Asian (0.03 difference in mean score) or Black (0.05 difference in mean score) physicians were less likely to rate their physicians favorably than their counterparts who saw White physicians; the latter difference was underpowered to reach statistical significance in adjusted analyses. Black patients who saw White (0.03 difference in mean score) or Asian (0.06 difference in mean score) physicians were less likely to rate their physicians favorably compared with those who saw Black physicians. To put these differences into perspective, in another study of the PG survey,^2^ a decrease in mean score of 0.02 on the 1-to-5 scoring scale was associated with a substantial decrease in physician ranking from the 100th to 70th percentile.

Our findings add to the existing literature by presenting new information that supports the positive associations between the patient experience as measured by the PG survey and patient-physician racial concordance. Although 2 prior studies^20,24^ evaluated PG scores in broad medical settings, the associations between physician ratings and patient and physician characteristics were mixed. These studies were limited by the patient populations included (predominantly White and Asian patients^24^ and inpatients^20^) and the exclusion of characteristics that we found to be important determinants of physician ratings. We evaluated patient-physician racial/ethnic dyads beyond those of White and Asian racial groups and accounted for a more comprehensive group of patient, physician, and visit characteristics. As such, our study lends further support to the clinical benefits that others have found to be associated with racially/ethnically concordant patient-physician interactions.^8,11,12,19^ These benefits include better patient-physician communication,^6,31,32^ patient care,^33^ and outcomes^34^ and have been suggested to be attributable to decreased bias between patients and physicians.^6,7^ Together, this information should be viewed as a call to action to vigorously support the training of underrepresented minority medical students and residents while also ensuring the promotion and retention of underrepresented minority physicians. However, the provision of health care to minority patients should not fall solely to minority physicians. It is, therefore, imperative that we also improve cultural mindfulness among all physicians so that they are prepared to care for a diverse patient population in an equitable manner.

We also identified other PG score patterns that raise questions about how these scores should be used. PG scores for physicians who saw White patients were numerically higher than for physicians who saw non-White patients. In adjusted analyses, Black and Asian patient race were each associated with lower odds of the physician receiving the maximum score compared with White patient race. These findings are consistent with and expand upon prior studies^24,35^ that observed that Asian patients rate their physicians lower than do White patients. We also found that, among all patients except for Black patients, the absolute proportion of physicians who received the maximum score was lowest among Black physicians. Furthermore, among White patients, the odds of receiving the maximum score were similar between White and Hispanic physicians, the latter of whom may, in some cases, be less obviously identifiable by White patients as being ethnically discordant. Although these patterns may reflect truly different patient experiences, they also raise the possibility that there are racial/ethnic differences in patient expectations of or biases toward their physicians that may influence PG scores. This is especially relevant in our current environment of #WhatADoctorLooksLike,^36^ in which examples of the lay public questioning the medical authority of underrepresented minority physicians are too common. Thus, care should be taken in publicly reporting individual PG scores or using them for physician compensation and promotion decisions so as not to disincentivize physicians from caring for a diverse patient population or accelerate job dissatisfaction.^1,37^

With respect to gender, in contrast to some studies, we did not find an association between patient-physician gender concordance and PG scores. As suggested in prior work,^10^ it is possible that associations between patient-physician gender concordance and patient experience ratings are particular to specific medical specialties.

### Strengths and Limitations

Study strengths include a large survey sample; robust patient-level, physician-level, and visit-level data; examination of all Care Provider domain PG survey questions; and inclusion of a racially/ethnically diverse patient population. Limitations include the low survey response rate and the potential for nonresponse bias, small numbers among some racial/ethnic groups (especially Black and Hispanic physicians), and lack of generalizability to patient and physician populations with different sociodemographic compositions. The survey response rate for our study is typical of the PG survey, as has been reported in other literature,^2,10,20,24,38^ and, although it is beyond the scope of the present study, the presence of nonresponse bias has been suggested in at least one study of the PG survey.^38^ Nonresponse bias may contribute to skewed responses in both the positive and negative directions. Future studies aimed at quantifying the effects of nonresponse bias are necessary for appropriate interpretation of PG scores.

## Conclusions

This cross-sectional analysis provides new insights about the associations between the patient experience as measured by PG scores and patient-physician racial/ethnic concordance as well as patient race/ethnicity. Further investigation to understand the reasons for differential scoring across specific patient and physician characteristics is warranted. In the meantime, although we recognize the value of PG scores as a measure of the patient experience, we also encourage health care leaders, administrators, and insurers to reevaluate the appropriateness of using PG scores as a primary and reportable measure of physician performance on an individual level.

## Notes

Supplement. **eAppendix.** Patient Survey **eFigure 1.** Study Selection Flow Diagram **eFigure 2.** Adjusted Odds Ratios for Secondary Patient Experience Outcomes by Patient-Physician Gender Discordant Pairs **eTable 1.** Patient Characteristics by Physician Race/Ethnicity **eTable 2.** Adjusted Odds Ratios for Secondary Patient Experience Outcomes by Patient-Physician Racial/Ethnic Discordant Pairs **eTable 3.** Primary Model for Patient-Physician Racial/Ethnic Binary Concordance **eTable 4.** Primary Model for Patient-Physician Gender Binary Concordance
