# Supplementary material for: Association of Racial/Ethnic and Gender Concordance Between Patients and Physicians With Patient Experience Ratings
Source: JAMA Netw Open. 2020 Nov 9;3(11):e2024583. doi: 10.1001/jamanetworkopen.2020.24583 (PMC7653497; doi:10.1001/jamanetworkopen.2020.24583)
Supplement: Supplement. — eAppendix. Patient Survey eFigure 1. Study Selection Flow Diagram eFigure 2. Adjusted Odds Ratios for Secondary Patient Experience Outcomes by Patient-Physician Gender Discordant Pairs eTable 1. Patient Characteristics by Physician Race/Ethnicity eTable 2. Adjusted Odds Ratios for Secondary Patient Experience Outcomes by Patient-Physician Racial/Ethnic Discordant Pairs eTable 3. Primary Model for Patient-Physician Racial/Ethnic Binary Concordance eTable 4. Primary Model for Patient-Physician Gender Binary Concordance [file jamanetwopen-e2024583-s001.pdf]

Supplemental Online Content

Takeshita J, Wang S, Loren AW, et al. Association of racial/ethnic and gender concordance between patients and physicians with patient experience ratings. *JAMA Netw Open*. 2020;3(11):e2024583. doi:10.1001/jamanetworkopen.2020.24583

eAppendix. Patient Survey

eFigure 1. Study Selection Flow Diagram

eFigure 2. Adjusted Odds Ratios for Secondary Patient Experience Outcomes by Patient-Physician Gender Discordant Pairs

eTable 1. Patient Characteristics by Physician Race/Ethnicity

eTable 2. Adjusted Odds Ratios for Secondary Patient Experience Outcomes by Patient-Physician Racial/Ethnic Discordant Pairs

eTable 3. Primary Model for Patient-Physician Racial/Ethnic Binary Concordance

eTable 4. Primary Model for Patient-Physician Gender Binary Concordance

This supplemental material has been provided by the authors to give readers additional information about their work.

© 2020 Takeshita J et al. *JAMA Network Open.*

eAppendix. Patient Survey

CLINICIAN AND GROUP EXPERIENCE SURVEY

SURVEY INSTRUCTIONS: Answer each question by completely filling in the circle to the left of your answer. You are sometimes told to skip over some questions in this survey. When this happens you will see an arrow with a note that tells you what question to answer next.

YOUR PROVIDER

1. Our records show that you visited the provider named below
   Precode 3
   Is that right?
   - o Yes
   - o No → **If No, please stop and return the survey in the envelope provided.**

The questions in this survey will refer to the provider named in Question 1 as "this provider." Please think of that person as you answer the survey.

2. Is this the provider you usually see if you need a check-up, want advice about a health problem, or get sick or hurt?
   - o Yes
   - o No

Questions that ask about "this visit" are referring to your visit with this provider on Precode 1

APPOINTMENT AND OFFICE CONTACT

3. Was this visit with this provider an appointment for an illness, injury or condition that **needed care right away**?
   - o Yes
   - o No → **If No, go to #5**

4. When you made this appointment for **care you needed right away**, did you get this appointment as soon as you thought you needed?
   - o Yes
   - o No

5. Was this visit with this provider an appointment **for a check-up or routine care**?
   - o Yes
   - o No → **If No, go to #7**

6. When you made this appointment **for a check-up or routine care**, did you get this appointment as soon as you thought you needed?
   - o Yes
   - o No

7. In the last 3 months, did you phone this provider's office with a medical question during regular office hours?
   - o Yes
   - o No → **If No, go to #9**

8. In the last 3 months, when you phoned this provider's office during regular office hours, how often did you get an answer to your medical question that same day?
   - o Never
   - o Sometimes
   - o Usually
   - o Always

9. In the last 3 months, did you phone this provider's office with a medical question **after** regular office hours?
   - o Yes
   - o No → **If No, go to #11**

10. In the last 3 months, when you phoned this provider's office **after** regular office hours, how often did you get an answer to your medical question as soon as you needed?
    - o Never
    - o Sometimes
    - o Usually
    - o Always

11. In the last 3 months, did this provider order a blood test, x-ray, or other test for you?
    - o Yes
    - o No → **If No, go to #13**

12. In the last 3 months, when this provider ordered a blood test, x-ray, or other test for you, how often did someone from this provider's office follow-up to give you the results?
    - o Never
    - o Sometimes
    - o Usually
    - o Always

Continued...

© 2020 Takeshita J et al. *JAMA Network Open.*

YOUR CARE FROM THIS PROVIDER ON Precode 2

13. Wait time includes time spent in the waiting room and exam room. During this visit, did you see this provider **within 15 minutes** of your appointment time?
    - o Yes
    - o No

14. During this visit, did this provider explain things in a way that was easy to understand?
    - o Yes, definitely
    - o Yes, somewhat
    - o No

15. During this visit, did this provider listen carefully to you?
    - o Yes, definitely
    - o Yes, somewhat
    - o No

16. During this visit, did you talk with this provider about any health questions or concerns?
    - o Yes
    - o No → **If No, go to #18**

17. During this visit, did this provider give you easy to understand information about these health questions or concerns?
    - o Yes, definitely
    - o Yes, somewhat
    - o No

18. During this visit, did this provider seem to know the important information about your medical history?
    - o Yes, definitely
    - o Yes, somewhat
    - o No

19. During this visit, did this provider have your medical records?
    - o Yes
    - o No

20. During this visit, did this provider show respect for what you had to say?
    - o Yes, definitely
    - o Yes, somewhat
    - o No

21. During this visit, did this provider spend enough time with you?
    - o Yes, definitely
    - o Yes, somewhat
    - o No

22. Using any number from 1 to 10, where 0 is the worst provider possible and 10 is the best provider possible. What number would you use to rate this provider?
    - o 0 Worst provider possible
    - o 1
    - o 2
    - o 3
    - o 4
    - o 5
    - o 6
    - o 7
    - o 8
    - o 9
    - o 10 Best provider possible

23. Would you recommend this provider's office to your family and friends?
    - o Yes, definitely
    - o Yes, somewhat
    - o No

CLERKS AND RECEPTIONISTS AT THIS PROVIDER'S OFFICE

24. During this visit, were clerks and receptionists at this provider's office as helpful as you thought they should be?
    - o Yes, definitely
    - o Yes, somewhat
    - o No

25. During this visit, did clerks and receptionists at this provider's office treat you with courtesy and respect?
    - o Yes, definitely
    - o Yes, somewhat
    - o No

ALL YOUR CARE IN THE LAST 3 MONTHS

These questions ask about all your health care. Include all the providers you saw for health care in the last 3 months. Do not include the times you saw a dentist.

26. In the last 3 months, did you take any prescription medicine?
    - o Yes
    - o No → **If No, go to #28**

27. In the last 3 months, how often did you and anyone on your health care team talk about all the prescription medicines you were taking?
    - o Never
    - o Sometimes
    - o Usually
    - o Always

Continued...

© 2020 Takeshita J et al. *JAMA Network Open.*

ABOUT YOU

28. In general, how would you rate your overall health?
    - o Excellent
    - o Very good
    - o Good
    - o Fair
    - o Poor

29. In general, how would you rate your overall **mental or emotional** health?
    - o Excellent
    - o Very good
    - o Good
    - o Fair
    - o Poor

30. What is the highest grade or level of school that you have completed?
    - o 8th grade or less
    - o Some high school, but did not graduate
    - o High school graduate or GED
    - o Some college or 2-year degree
    - o 4-year college graduate
    - o More than 4-year college degree

31. Are you of Hispanic, Latino, or Spanish origin?
    - o Yes, Hispanic, Latino, or Spanish
    - o No, not Hispanic, Latino, or Spanish

32. What is your race? Mark one or more.
    - o White
    - o Black or African American
    - o Asian
    - o Native Hawaiian or Other Pacific Islander
    - o American Indian or Alaskan Native
    - o Other ________________________ (specify)

33. Did someone help you complete this survey?
    - o Yes
    - o No → **If No, go to ADDITIONAL QUESTIONS ABOUT THIS VISIT**

34. How did that person help you? Mark one or more.
    - o Read the questions to me
    - o Wrote down the answers I gave
    - o Answered the questions for me
    - o Translated the questions into my language
    - o Helped in some other way
      Please print: ____________________

PRESS GANEY SURVEY

Now that we have asked you to tell us about *what happened* during your recent experience with the provider and his/her office, please rate how satisfied you were with the services you received during this visit. INSTRUCTIONS: Mark the response that best describes your experience. If a question does not apply to you, please skip to the next question. Space is provided for you to comment on your experiences.

**ACCESS**	**Very Poor 1**	**Poor 2**	**Fair 3**	**Good 4**	**Very Good 5**
1. Ease of scheduling your appointment ……………………………….	O	O	O	O	O
2. Courtesy of person who scheduled your appointment …………….	O	O	O	O	O
3. Ability of getting an appointment within a reasonable time frame...	O	O	O	O	O
4. Ease of getting through to the practice on the phone ……………..	O	O	O	O	O
5. Our helpfulness on the telephone …………………………………..	O	O	O	O	O
6. Our promptness in returning your phone calls ……………………..	O	O	O	O	O
7. Convenience of our office hours ……………………………………..	O	O	O	O	O
8. Courtesy of staff in the registration area …………………………….	O	O	O	O	O

Comments (describe good or bad experience): ____

____

**MOVING THROUGH YOUR VISIT**	**Very Poor 1**	**Poor 2**	**Fair 3**	**Good 4**	**Very Good 5**
1. Comfort and pleasantness of the reception area …………………..	O	O	O	O	O
2. Comfort and pleasantness of the exam room ………………………	O	O	O	O	O
3. Degree to which you were informed about any delays ……………	O	O	O	O	O

this section continued on next page…

© 2020 Takeshita J et al. *JAMA Network Open.*

MOVING THROUGH YOUR VISIT (…continued)	Very Poor 1	Poor 2	Fair 3	Good 4	Very Good 5
4. Wait time at clinic (from arriving to leaving) …………………….....	O	O	O	O	O

Comments (describe good or bad experience): ____
____

NURSE/ASSISTANT	Very Poor 1	Poor 2	Fair 3	Good 4	Very Good 5
1. Friendliness/courtesy of nurse/assistant …………………………...	O	O	O	O	O
2. Concern the nurse/assistant showed for your problem …………..	O	O	O	O	O

Comments (describe good or bad experience): ____
____

CARE PROVIDER<br>**Please answer these questions with the Provider named in the first question of this survey in mind**	Very Poor 1	Poor 2	Fair 3	Good 4	Very Good 5
1. Friendliness/courtesy of the care provider …………………………	O	O	O	O	O
2. Explanations the care provider gave you about your problem or condition …………………………………………………………...	O	O	O	O	O
3. Concern the care provider showed for your questions or worries ..	O	O	O	O	O
4. Care provider's efforts to include you in decisions about your treatment …………………………………………………………..	O	O	O	O	O
5. Information the care provider gave you about medications (if any).	O	O	O	O	O
6. Instructions the care provider gave you about follow-up care (if any) …………………………………………………………………	O	O	O	O	O
7. Degree to which care provider talked with you using words you could understand …………………………………………………….	O	O	O	O	O
8. Amount of time the care provider spent with you …………………..	O	O	O	O	O
9. Your confidence in this care provider ………………………………..	O	O	O	O	O
10. Likelihood of your recommending this care provider to others ……	O	O	O	O	O

Comments (describe good or bad experience): ____
____

PERSONAL ISSUES	Very Poor 1	Poor 2	Fair 3	Good 4	Very Good 5
1. How well staff protected your safety (washing hands, wearing gloves, etc.) ……………………………………………………………	O	O	O	O	O
2. Our sensitivity to your needs …………………………………………	O	O	O	O	O
3. Our concern for your privacy ………………………………………...	O	O	O	O	O
4. Cleanliness of our practice …………………………………………...	O	O	O	O	O
5. Degree to which staff cleaned (gel or soap) their hands before and after caring for you …………………………………………….....	O	O	O	O	O

Comments (describe good or bad experience): ____
____

OVERALL ASSESSMENT	Very Poor 1	Poor 2	Fair 3	Good 4	Very Good 5
1. How well the staff worked together to care for you ………………..	O	O	O	O	O
2. Overall rating of your experience during your visit …………………	O	O	O	O	O

this section continued on next page…

© 2020 Takeshita J et al. *JAMA Network Open.*

OVERALL ASSESSMENT (… continued)	Very Poor 1	Poor 2	Fair 3	Good 4	Very Good 5
3. Likelihood of your recommending our practice to others …………	O	O	O	O	O

Comments (describe good or bad experience): ____
____

Patient's Name: (optional) ____

Telephone Number: (optional) ____

© 2020 Takeshita J et al. *JAMA Network Open.*

eFigure 1. Study Selection Flow Diagram

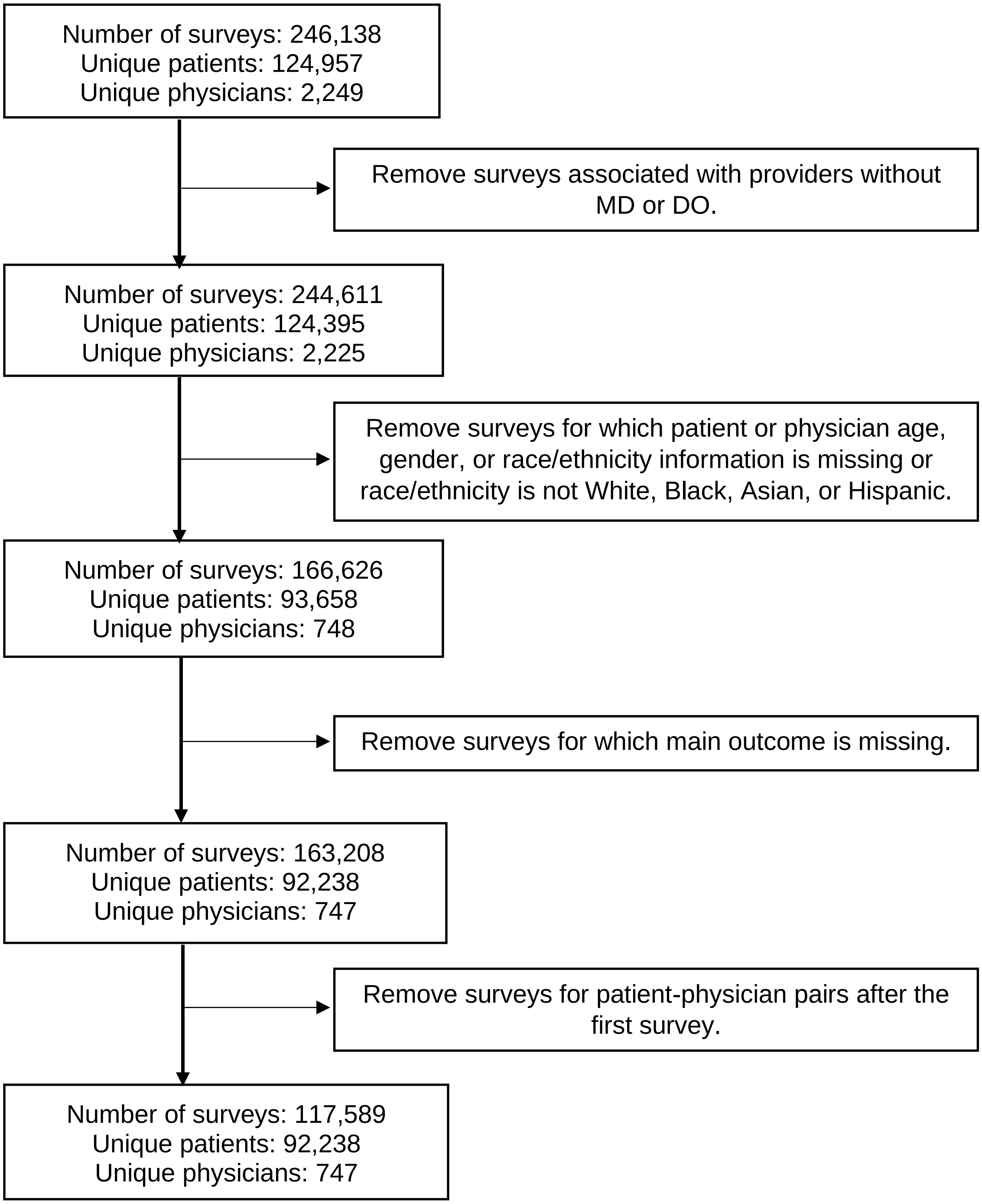

© 2020 Takeshita J et al. *JAMA Network Open.*

eFigure 2. Adjusted Odds Ratios for Secondary Patient Experience Outcomes by Patient-Physician Gender Discordant Pairs

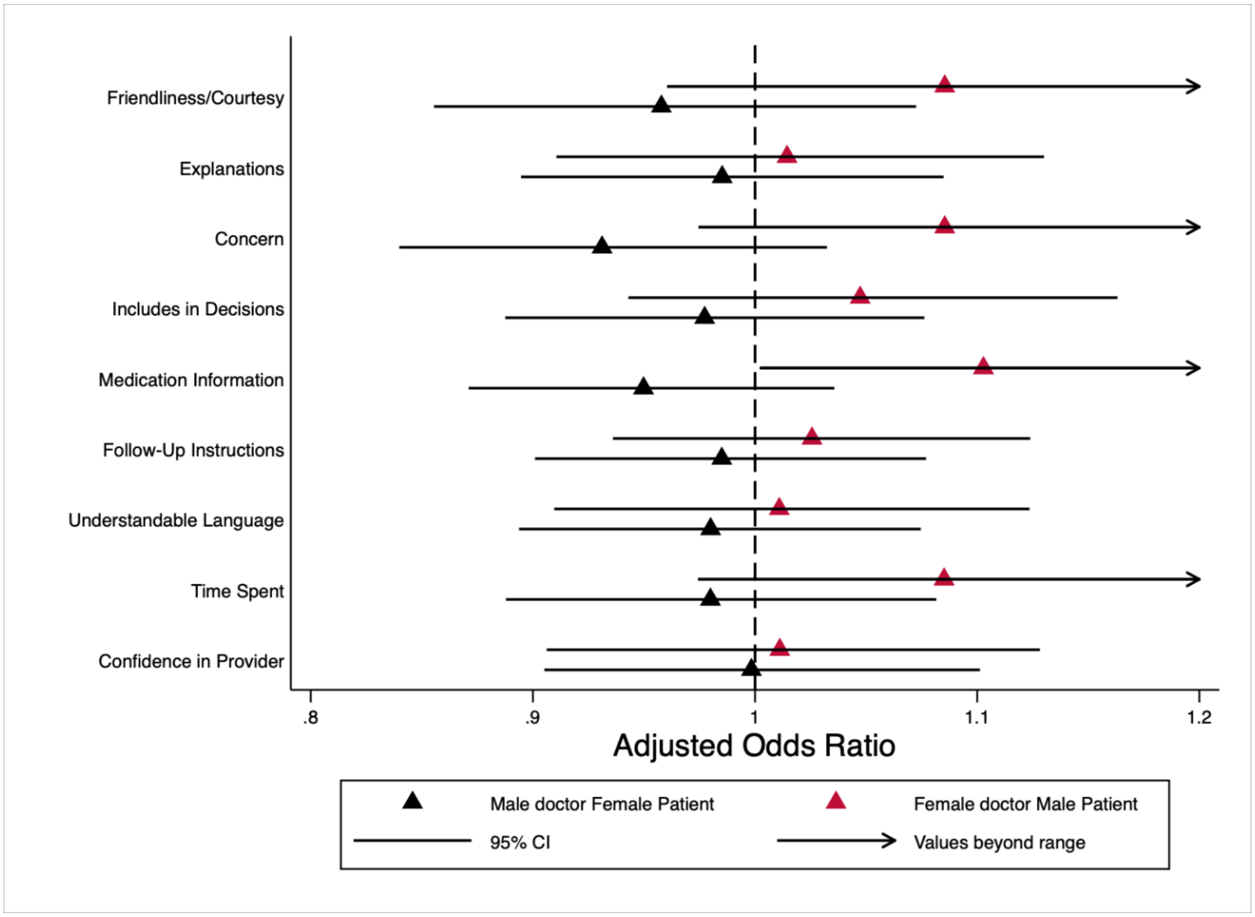

Gender concordance regression models included the following variables: patient age, gender, race/ethnicity, education, income, marital status, insurance, primary language, overall health, and mental or emotional health; physician age, gender, race/ethnicity, academic track, and academic rank; and clinical counter type, location, and specialty. Gender conordant patient-physician pairs serve as the reference.

© 2020 Takeshita J et al. *JAMA Network Open.*

eTable 1. Patient Characteristics by Physician Race/Ethnicity

Patient Characteristics	White Physician	Black Physician	Asian Physician	Hispanic Physician	All Physicians
**Total no. of patients**	73,405	5,063	19,619	2,495	100,582
**No. of unique patient surveys per physician**					
Mean (SD)	167.5 (174.0)	151.4 (149.8)	131.6 (124.0)	109.0 (86.6)	157.4 (162.2)
Median (interquartile range)	126 (50, 220)	121.5 (44, 206)	97 (41, 194)	94 (36, 183)	114 (46, 213)
**Age**					
Mean (SD)	58.3 (15.2)	54.3 (16.0)	57.3 (15.9)	55.0 (16.7)	57.9 (15.5)
Median (interquartile range)	61 (49, 69)	57 (41, 67)	60 (47, 69)	58 (40, 68)	60 (48, 69)
**Gender**					
Male	29,960 (40.8)	1,314 (26.0)	7,810 (39.8)	989 (39.6)	40,073 (39.8)
**Race/Ethnicity**					
White	61,026 (83.1)	3,501 (69.1)	15,667 (79.9)	1,919 (76.9)	82,113 (81.6)
Black	8,499 (11.6)	1,265 (25.0)	2,734 (13.9)	386 (15.5)	12,884 (12.8)
Asian	2,291 (3.1)	164 (3.2)	784 (4.0)	102 (4.1)	3,341 (3.3)
Hispanic	1,589 (2.2)	133 (2.6)	434 (2.2)	88 (3.5)	2,244 (2.2)
**Marital Status**					
Single	16,299 (22.2)	1,434 (28.3)	4,635 (23.6)	639 (25.6)	23,007 (22.9)
Married or with a partner	48,075 (65.5)	2,976 (58.8)	12,416 (63.3)	1,576 (63.2)	65,043 (64.7)
Divorced or widowed	8,714 (11.9)	632 (12.5)	2,478 (12.6)	267 (10.7)	12,091 (12.0)
Other	317 (0.43)	21 (0.41)	90 (0.46)	13 (0.52)	441 (0.44)
**Insurance**					
Commercial	39,120 (53.3)	2,973 (58.7)	10,512 (53.6)	1,359 (54.5)	53,964 (53.7)
Medicare	24,878 (33.9)	1,359 (26.8)	6,337 (32.3)	774 (31.0)	33,348 (33.2)
Medicaid	2,534 (3.5)	300 (5.9)	932 (4.8)	130 (5.2)	3,896 (3.9)
Tricare or Veterans Affairs	440 (0.60)	23 (0.45)	153 (0.78)	18 (0.72)	634 (0.63)
Workers' compensation	307 (0.42)	19 (0.38)	61 (0.31)	9 (0.36)	396 (0.39)
Mixed	4,303 (5.9)	293 (5.8)	1,105 (5.6)	140 (5.6)	5,841 (5.8)
Other	1,823 (2.5)	96 (1.9)	519 (2.6)	65 (2.6)	2,503 (2.5)
**Education**					
Up to high school or general education diploma	11,780 (16.0)	765 (15.1)	3,528 (18.0)	411 (16.5)	16,484 (16.4)

© 2020 Takeshita J et al. *JAMA Network Open.*

Some college	16,208 (22.1)	1,166 (23.0)	4,412 (22.5)	565 (22.6)	22,351 (22.2)
4-y College graduate	15,996 (21.8)	1,077 (21.3)	4,163 (21.2)	522 (20.9)	21,758 (21.6)
More than 4-y college graduate	28,660 (39.0)	2,008 (39.7)	7,287 (37.1)	973 (39.0)	38,928 (38.7)
Missing	761 (1.0)	47 (0.93)	229 (1.2)	24 (0.96)	1,061 (1.1)
**Median Annual Household Income, $**					
Mean (SD)	80,028 (31,572)	72,290 (31,820)	76,800 (31,076)	73,774 (30,426)	78,853 (31,533)
Median (interquartile range)	77,303 (58,784, 99,167)	70,638 (49,329, 94,313)	73,848 (55,481, 95,537)	71,281 (52,647, 93,497)	76,464 (56,634, 97,452)
**Primary Language**					
English	72,752 (99.1)	5,018 (99.1)	19,407 (98.9)	2,466 (98.8)	99,643 (99.1)
**Survey Assistance**					
None	70,518 (96.1)	4,913 (97.0)	18,626 (94.9)	2,362 (94.7)	96,419 (95.9)
Answered for me	933 (1.3)	50 (0.99)	363 (1.9)	37 (1.5)	1,383 (1.4)
Wrote answers	501 (0.68)	31 (0.61)	167 (0.85)	28 (1.1)	727 (0.72)
Read questions	409 (0.56)	28 (0.55)	148 (0.75)	24 (0.96)	609 (0.61)
Translation	80 (0.11)	2 (0.04)	38 (0.19)	3 (0.12)	123 (0.12)
Other	185 (0.25)	7 (0.14)	58 (0.30)	13 (0.52)	263 (0.26)
Missing	779 (1.1)	32 (0.63)	219 (1.1)	28 (1.1)	1,058 (1.1)
**Overall Health**					
Poor	2,169 (3.0)	134 (2.6)	660 (3.4)	106 (4.2)	3,069 (3.05)
Fair	10,084 (13.7)	734 (14.5)	2,825 (14.4)	410 (16.4)	14,053 (14.0)
Good	23,489 (32.0)	1,626 (32.1)	6,469 (33.0)	880 (35.3)	32,464 (32.3)
Very good	26,440 (36.0)	1,839 (36.3)	6,979 (35.6)	787 (31.5)	36,045 (35.8)
Excellent	10,792 (14.7)	700 (13.8)	2,561 (13.0)	296 (11.9)	14,349 (14.3)
Missing	431 (0.59)	30 (0.59)	125 (0.64)	16 (0.64)	602 (0.60)
**Overall Mental or Emotional Health**					
Poor	966 (1.3)	76 (1.5)	297 (1.5)	48 (1.9)	1,387 (1.4)
Fair	5,953 (8.1)	465 (9.2)	1,751 (8.9)	278 (11.1)	84,47 (8.4)
Good	15,565 (21.2)	1,159 (22.9)	4,412 (22.5)	597 (23.9)	21,733 (21.6)
Very good	25,390 (34.6)	1,819 (35.9)	6,844 (34.9)	865 (34.7)	34,918 (34.7)
Excellent	25,119 (34.2)	1,514 (29.9)	6,197 (31.6)	698 (28.0)	33,528 (33.3)
Missing	412 (0.56)	30 (0.59)	118 (0.60)	9 (0.36)	569 (0.57)

© 2020 Takeshita J et al. *JAMA Network Open.*

eTable 2. Adjusted Odds Ratios for Secondary Patient Experience Outcomes by Patient-Physician Racial/Ethnic Discordant Pairs

Secondary Outcomes (survey questions)	Patient	Physician			
		White	Black	Asian	Hispanic
Friendliness/Courtesy	White	reference	0.83 (0.60, 1.15)	0.86 (0.74, 0.98)	1.01 (0.74, 1.38)
	Black	0.86 (0.65, 1.15)	reference	0.81 (0.60, 1.10)	1.09 (0.71, 1.67)
	Asian	1.15 (0.94, 1.41)	0.82 (0.58, 1.17)	reference	1.02 (0.61, 1.70)
	Hispanic	1.06 (0.66, 1.70)	0.94 (0.44, 2.00)	0.91 (0.54, 1.53)	reference
Explanations	White	reference	0.79 (0.62, 1.03)	0.86 (0.76, 0.96)	1.03 (0.81, 1.30)
	Black	0.87 (0.68, 1.12)	reference	0.78 (0.60, 1.02)	1.03 (0.72, 1.50)
	Asian	1.17 (0.96, 1.43)	1.00 (0.71, 1.41)	reference	1.64 (0.94, 2.85)
	Hispanic	1.01 (0.59, 1.72)	0.72 (0.37, 1.37)	0.84 (0.48, 1.49)	reference
Concern	White	reference	0.80 (0.60, 1.06)	0.83 (0.74, 0.94)	1.07 (0.81, 1.41)
	Black	0.83 (0.63, 1.11)	reference	0.69 (0.51, 0.93)	0.93 (0.62, 1.38)
	Asian	1.06 (0.87, 1.29)	0.89 (0.61, 1.28)	reference	1.53 (0.94, 2.48)
	Hispanic	1.03 (0.65, 1.65)	0.90 (0.48, 1.68)	0.91 (0.55, 1.52)	reference
Includes in Decisions	White	reference	0.83 (0.64, 1.08)	0.81 (0.72, 0.91)	1.05 (0.81, 1.36)
	Black	0.80 (0.60, 1.07)	reference	0.65 (0.48, 0.89)	0.87 (0.57, 1.34)
	Asian	1.08 (0.88, 1.34)	0.87 (0.59, 1.28)	reference	1.24 (0.75, 2.04)
	Hispanic	1.05 (0.63, 1.72)	0.90 (0.48, 1.68)	0.87 (0.51, 1.49)	reference
Medication Information	White	reference	0.84 (0.68, 1.04)	0.86 (0.78, 0.96)	1.00 (0.79, 1.27)
	Black	0.85 (0.66, 1.10)	reference	0.75 (0.57, 0.98)	0.91 (0.59, 1.39)
	Asian	1.08 (0.87, 1.35)	0.97 (0.66, 1.41)	reference	1.28 (0.81, 2.03)
	Hispanic	1.07 (0.63, 1.83)	0.96 (0.51, 1.79)	1.01 (0.57, 1.77)	reference
Follow-Up Instructions	White	reference	0.81 (0.64, 1.02)	0.85 (0.77, 0.94)	1.07 (0.87, 1.32)
	Black	0.93 (0.75, 1.15)	reference	0.80 (0.64, 1.01)	1.07 (0.74, 1.54)
	Asian	1.08 (0.87, 1.33)	1.03 (0.69, 1.54)	reference	1.20 (0.70, 2.06)
	Hispanic	1.294 (0.81, 2.07)	1.20 (0.63, 2.28)	1.14 (0.69, 1.91)	reference
Understandable Language	White	reference	0.86 (0.67, 1.11)	0.85 (0.76, 0.95)	1.15 (0.88, 1.50)
	Black	0.82 (0.64, 1.07)	reference	0.75 (0.57, 0.98)	0.93 (0.65, 1.34)
	Asian	1.07 (0.87, 1.32)	0.78 (0.55, 1.10)	reference	1.22 (0.58, 2.55)
	Hispanic	1.08 (0.62, 1.88)	0.88 (0.41, 1.88)	1.01 (0.56, 1.81)	reference
Time Spent	White	reference	0.93 (0.72, 1.21)	0.85 (0.76, 0.95)	1.04 (0.82, 1.32)
	Black	0.75 (0.57, 0.98)	reference	0.64 (0.48, 0.85)	0.87 (0.62, 1.22)
	Asian	1.15 (0.95, 1.40)	1.13 (0.81, 1.59)	reference	1.41 (0.83, 2.40)
	Hispanic	0.99 (0.61, 1.59)	0.78 (0.41, 1.49)	0.73 (0.44, 1.22)	reference

© 2020 Takeshita J et al. *JAMA Network Open.*

Confidence in Provider	White	reference	0.84 (0.64, 1.20)	0.88 (0.79, 0.98)	1.06 (0.83, 1.35)
	Black	0.79 (0.61, 1.02)	reference	0.68 (0.52, 0.90)	0.78 (0.54, 1.13)
	Asian	1.04 (0.87, 1.26)	0.78 (0.54, 1.12)	reference	1.19 (0.82, 1.74)
	Hispanic	0.94 (0.57, 1.53)	0.68 (0.34, 1.37)	0.89 (0.52, 1.50)	reference

Patient-physician racial/ethnic concordance regression models included the following variables: patient age, gender, race/ethnicity, education, income, marital status, insurance, primary language, overall health, and mental or emotional health; physician gender, race/ethnicity, and academic rank; and clinical counter type, location, and specialty.

© 2020 Takeshita J et al. *JAMA Network Open.*

eTable 3. Primary Model for Patient-Physician Racial/Ethnic Binary Concordance

	Odds Ratio	95% Conf. Interval		P-value
**Patient-Physician Racial/Ethnic Concordance**				
Concordant	reference			
Discordant	0.88	0.82	0.94	<0.001
**Patient Race/Ethnicity**				
White	reference			
Black	0.73	0.68	0.78	<0.001
Asian	0.55	0.50	0.60	<0.001
Hispanic	0.92	0.81	1.04	0.18
**Patient Age**	1.01	1.01	1.02	<0.001
**Patient Gender**				
Female	reference			
Male	1.06	1.02	1.10	0.005
**Patient Education**				
Up to high school or general education diploma	reference			
Some college	1.01	0.96	1.07	0.69
4-y College graduate	0.87	0.82	0.92	<0.001
More than 4-y college graduate	0.83	0.79	0.88	<0.001
**Patient Income per $10,000**	1.01	1.00	1.01	0.13
**Patient Marital Status**				
Single	reference			
Married or with a partner	1.19	1.14	1.25	<0.001
Divorced or widowed	1.07	1.01	1.14	0.02
Other	1.07	0.84	1.35	0.59
**Patient Insurance**				
Commercial	reference			
Medicare	1.08	1.03	1.13	0.001
Medicaid	1.24	1.13	1.37	<0.001
Tricare or Veterans Affairs	1.10	0.89	1.36	0.37
Workers' compensation	1.34	1.05	1.72	0.02
Mixed	1.17	1.08	1.26	<0.001
Other	1.09	0.97	1.22	0.15
**Patient Language English**				
Yes	reference			
No	0.79	0.68	0.92	0.002
**Patient Overall Health**[a]	1.21	1.18	1.23	<0.001
**Patient Mental or Emotional Health**[a]	1.29	1.26	1.31	<0.001
**Physician Age**	0.99	0.98	1.00	0.003
**Physician Gender**				
Female	reference			
Male	1.02	0.93	1.13	0.63
**Physician Rank**				
Assistant professor	reference			
Associate professor	1.11	1.00	1.23	0.04

© 2020 Takeshita J et al. *JAMA Network Open.*

Professor	1.33	1.14	1.54	<0.001
Other	0.84	0.71	1.00	0.05
**Visit Type**				
New	reference			
Return	1.23	1.17	1.29	<0.001
Procedure	1.53	1.23	1.90	<0.001
**Location**				
Main	reference			
Affiliated – Philadelphia	0.89	0.83	0.95	0.001
Satellite – Pennsylvania	0.87	0.81	0.94	0.001
Satellite – New Jersey	0.95	0.76	1.19	0.67
**Specialty**				
Medical	reference			
Surgical	0.73	0.65	0.83	<0.001
Dermatology	0.80	0.64	0.99	0.04
Other	0.85	0.76	0.95	0.004
**Constant**	0.90	0.67	1.21	0.49

[a]Categorical variables analyzed as continuous (1 = poor health, 2 = fair health, 3 = good health, 4 = very good health, 5 = excellent health)

© 2020 Takeshita J et al. *JAMA Network Open.*

eTable 4. Primary Model for Patient-Physician Gender Binary Concordance

	Odds Ratio	95% Conf. Interval		P-value
**Patient-Physician Gender Concordance**				
Yes	reference			
No	1.00	0.96	1.04	0.13
**Patient Race/Ethnicity**				
White	reference			
Black	0.67	0.63	0.71	<0.001
Asian	0.51	0.47	0.55	<0.001
Hispanic	0.84	0.75	0.94	0.002
**Patient Age**	1.01	1.01	1.02	<0.001
**Patient Gender**				
Female	reference			
Male	1.01	1.01	1.02	0.004
**Patient Education**				
Up to high school or general education diploma	reference			
Some college	1.01	0.96	1.07	0.71
4-y College graduate	0.87	0.82	0.92	<0.001
More than 4-y college graduate	0.83	0.79	0.88	<0.001
**Patient Income per $10,000**	1.01	1.00	1.01	0.117
**Patient Marital Status**				
Single	reference			
Married or with a partner	1.19	1.14	1.25	<0.001
Divorced or widowed	1.07	1.01	1.14	0.02
Other	1.07	0.84	1.35	0.59
**Patient Insurance**				
Commercial	reference			
Medicare	1.08	1.03	1.14	0.001
Medicaid	1.24	1.13	1.37	<0.001
Tricare or Veterans Affairs	1.10	0.89	1.35	0.39
Workers' compensation	1.33	1.04	1.72	0.02
Mixed	1.17	1.08	1.26	<0.001
Other	1.09	0.97	1.23	0.13
**Patient Language English**				
Yes	reference			
No	0.79	0.68	0.92	0.003
**Patient Overall Health[a]**	1.21	1.18	1.23	<0.001
**Patient Mental or Emotional Health[a]**	1.29	1.26	1.31	<0.001
**Physician Age**	0.99	0.98	1.00	0.02
**Physician Race/Ethnicity**				
White	reference			
Black	0.99	0.78	1.26	0.96
Asian	0.88	0.80	0.98	0.02
Hispanic	1.03	0.83	1.28	0.78

© 2020 Takeshita J et al. *JAMA Network Open.*

**Physician Track**				
Academic clinician	reference			
Clinical	0.95	0.81	1.12	0.56
Clinician Educator	1.06	0.95	1.18	0.27
Tenure	0.90	0.72	1.13	0.36
Others	0.85	0.72	1.01	0.06
**Physician Rank**				
Assistant professor	reference			
Associate professor	1.10	1.00	1.22	0.06
Professor	1.34	1.15	1.56	<0.001
Other	0.84	0.71	0.99	0.04
**Physician Track**				
Academic clinician	reference			
Clinical	0.95	0.81	1.12	0.56
Clinician Educator	1.06	0.95	1.18	0.27
Tenure	0.90	0.72	1.13	0.36
Others	0.85	0.72	1.01	0.06
**Visit Type**				
New	reference			
Return	1.23	1.17	1.29	<0.001
Procedure	1.53	1.22	1.91	<0.001
**Location**				
Main	reference			
Affiliated – Philadelphia	0.90	0.83	0.97	0.005
Satellite – Pennsylvania	0.87	0.80	0.95	0.001
Satellite – New Jersey	0.96	0.77	1.20	0.71
**Specialty**				
Medical	reference			
Surgical	0.73	0.65	0.82	<0.001
Dermatology	0.81	0.65	1.00	0.05
Other	0.83	0.74	0.93	0.001
**Constant**	0.85	0.62	1.18	0.34

[a]Categorical variables analyzed as continuous (1 = poor health, 2 = fair health, 3 = good health, 4 = very good health, 5 = excellent health)

© 2020 Takeshita J et al. *JAMA Network Open.*
